# Transcriptomic integration reveals immune heterogeneity in vascular dementia and identifies ELF2 as a candidate therapeutic target

**DOI:** 10.1097/MD.0000000000050324

**Published:** 2026-09-11

**Authors:** Li Liang, Cuiqin Shen, Shiren Huang, Minglu Hu, Jian Zhu

**Affiliations:** a Department of Neurology, Jiading Branch of Shanghai General Hospital, Shanghai Jiao Tong University School of Medicine, Shanghai, China; b Department of Ultrasound, Jiading Branch of Shanghai General Hospital, Shanghai Jiao Tong University School of Medicine, Shanghai, China.

**Keywords:** ELF2, immune heterogeneity, neuroglial–vascular unit, single-cell RNA sequencing, vascular dementia

## Abstract

Vascular dementia (VaD) is the second most prevalent form of dementia after Alzheimer’s disease and is primarily driven by chronic cerebral hypoperfusion and neurovascular dysfunction. Growing evidence indicates that immune dysregulation and neuroglial–vascular injury play critical roles in disease progression; however, the molecular regulators underlying these processes remain insufficiently characterized. This study aimed to delineate immune heterogeneity and neuroglial–vascular alterations in VaD and to identify key regulatory targets involved in disease pathogenesis. Bulk RNA sequencing data (GSE122063) and single-cell transcriptomic data (GSE282111) were retrieved from the Gene Expression Omnibus database and integratively analyzed to characterize immune infiltration patterns and cellular dysregulation. Immune profiling was performed using CIBERSORT and single-sample gene set enrichment analysis. Disease-associated cell subsets and hub genes were identified using Seurat, Scissor, and high-dimensional weighted gene co-expression network analysis (hdWGCNA). Pseudotime trajectory analysis and molecular docking were subsequently applied to investigate gene expression dynamics and potential therapeutic relevance. Bulk transcriptomic analysis revealed a pronounced pro-inflammatory shift in VaD brain tissue, characterized by increased infiltration of M1 macrophages, neutrophils, and activated dendritic cells, alongside reduced levels of M2 macrophages, resting CD4+ memory T cells, and regulatory T cells. Single-cell analysis demonstrated marked loss of oligodendrocytes, astrocytes, and endothelial cells in VaD. Scissor integration showed positive associations between VaD and oligodendrocytes, astrocytes, and endothelial cells, whereas microglia and oligodendrocyte progenitor cells were negatively associated. hdWGCNA identified 3 co-expression modules; intersection of the blue module with bulk differentially expressed genes highlighted DOCK3, ELF2, and Sin3A associated protein 25, with ELF2 uniquely co-expressed across relevant cell types. Pseudotime analysis indicated sustained downregulation of ELF2 during VaD-related cellular differentiation. Molecular docking analysis suggested strong binding affinities between ELF2 and nicergoline (−7.23 kcal/mol), nimodipine (−6.92 kcal/mol), and donepezil (−6.29 kcal/mol). This integrative transcriptomic study reveals disrupted immune balance and neuroglial–vascular cell homeostasis in vascular dementia, and prioritizes ELF2 as a candidate regulator associated with immune heterogeneity in VaD.

## 1. Background

Vascular dementia (VaD) ranks as the second most prevalent form of dementia following Alzheimer’s disease.^[1]^ It typically arises from cerebral hypoperfusion and hypoxia, manifesting as progressive cognitive impairment.^[2]^ The pathogenesis of VaD involves dysregulation within the neurovascular unit. Chronic cerebral hypoperfusion initially compromises brain endothelial cells, leading to blood–brain barrier (BBB) disruption and impaired cerebral blood flow regulation.^[3]^ This cascade triggers glial cell activation and fosters an inflammatory microenvironment.^[4]^ Consequently, white matter myelin, axons, and synapses sustain damage, forming the histological basis of cognitive deficits.^[5]^

Extensive research highlights neuroinflammation, precipitated by chronic ischemia, as a central pathological feature of VaD.^[6]^ This inflammatory response is predominantly mediated by resident immune cells of the central nervous system, namely glial cells, chiefly microglia and astrocytes.^[7]^ Their hyperactivation results in neuronal and glial injury.^[8]^ Increasing attention has been directed toward associated immune dysregulation. Under physiological conditions, microglia exhibit dual functional phenotypes: M1 (pro-inflammatory) and M2 (anti-inflammatory), maintaining a balance to clear pathological debris and promote tissue repair.^[9]^ However, in chronic ischemic encephalopathies such as VaD, a pronounced shift toward the M1 phenotype occurs, accompanied by a deficiency in M2 activity, perpetuating an uncontrolled inflammatory state.^[10]^ For instance, hypoperfusion-induced injury prompts microglia to predominantly adopt the M1 phenotype, releasing pro-inflammatory cytokines (e.g., interleukin-1 beta [IL-1β], tumor necrosis factor-alpha [TNF-α]) and reactive oxygen species.^[11]^ This disrupts BBB integrity and damages neurons and oligodendrocytes.^[12]^ Meanwhile, the diminished secretion of anti-inflammatory mediators by M2 microglia hinders effective inflammation resolution.^[13]^

Under sustained ischemic and hypoxic stress, aberrant activation of microglia and astrocytes upregulates pro-inflammatory cytokines (e.g., IL-1β, interleukin-6, TNF-α), adhesion molecules (e.g., intercellular adhesion molecule-1, vascular cell adhesion molecule-1), and matrix metalloproteinases (MMP-2/9), amplifying neuroinflammation and further compromising the BBB.^[14,15]^ Conversely, expression of anti-inflammatory cytokines (e.g., IL-4, IL-10) declines, indicating an inadequate endogenous anti-inflammatory response.^[16]^ Collectively, these alterations establish an inflammatory microenvironment characterized by immune imbalance in VaD brain tissue, widely regarded as a key driver of disease onset and progression.^[17]^

Beyond immune-mediated inflammation, the degeneration and functional impairment of glial and vascular cell lineages are hallmark features of VaD progression. First, oligodendrocyte loss and demyelination are ubiquitous in the white matter of VaD patients.^[18]^ Chronic hypoperfusion induces oxygen–glucose deprivation and an inflammatory milieu, suppressing the differentiation and maturation of oligodendrocyte precursor cells (OPCs).^[19]^ This promotes demyelination and impairs axonal conduction, ultimately undermining cognitive function.^[20]^ Studies indicate that reactive oxygen species and proteases released by hyperactivated microglia directly damage oligodendrocytes and impede remyelination.^[21]^ Second, astrocytic dysfunction is a prominent characteristic of VaD. Astrocytes are critical for maintaining BBB integrity and cerebral metabolic homeostasis.^[22]^ In ischemic conditions, their foot processes fragment, and aquaporin-4 expression decreases, disrupting neurovascular coupling.^[23]^ Exposure to abundant pro-inflammatory mediators may further induce astrocytic hypertrophy (gliosis) or functional decline.^[24]^ Lastly, brain endothelial cell injury and dysfunction persist throughout VaD. Highly susceptible to ischemia–reperfusion injury, these cells exhibit early declines in endothelium-dependent vasodilation and loss of tight junction proteins, increasing BBB permeability.^[25]^ This facilitates peripheral immune cell infiltration into the CNS, intensifying inflammation, and exposes neurons to deleterious agents, precipitating white matter lesions and synaptic degeneration.^[26]^

Thus, VaD pathogenesis reflects a complex interplay of immune, glial, and vascular factors. Chronic cerebrovascular insufficiency triggers an inflammatory cascade that progressively damages glial and endothelial cells, which, in turn, weakens neuronal support and vascular homeostasis, perpetuating a vicious cycle.^[27]^ Building on this foundation, the present study investigates the molecular signatures of immune imbalance and glial/vascular cell lineage alterations in VaD brain tissue. Utilizing bulk RNA sequencing data (GSE122063) and single-cell RNA sequencing data (GSE282111) from the Gene Expression Omnibus (GEO) database,^[28,29]^ we integrated transcriptomic profiles at both population and single-cell resolution to comprehensively elucidate: enhanced infiltration of pro-inflammatory immune cells and relative scarcity of anti-inflammatory cells in VaD brains; quantitative and functional abnormalities in oligodendrocytes, astrocytes, and brain endothelial cells; identification of cell subsets significantly correlated with the VaD phenotype using the Scissor algorithm^[30]^; detection of key regulatory molecules (e.g., transcription factor ELF2) through high-dimensional weighted gene co-expression network analysis (hdWGCNA) and differential expression analysis; and dynamic changes and potential regulatory roles of critical genes during glial cell development via pseudotime analysis. Through this multi-level, multi-scale integration, we aim to deepen the understanding of VaD pathogenesis and provide a foundation for identifying novel therapeutic targets, such as E74 like ETS transcription factor 2(ELF2).

## 2. Materials and methods

### 2.1. Data acquisition and preprocessing

This study was approved by the Ethics Committee of Jiading Branch of Shanghai General Hospital. Bulk RNA sequencing (RNA-seq) data for VaD patients and controls (GSE122063) and raw single-cell RNA-seq matrices (GSE282111) were downloaded from the GEO database (https://www.ncbi.nlm.nih.gov/geo/). Bulk data were background-corrected and normalized to generate an expression matrix for immune deconvolution. Single-cell data were processed in Seurat: cells expressing 200 to 5000 genes with mitochondrial gene percentages < 10% were retained; UMI counts were normalized using the LogNormalize method, and following identification of highly variable genes, Harmony (v1.2.0; R package, Korsunsky et al, Broad Institute of MIT and Harvard) was used to correct batch effects among individual samples. Batch correction performance was evaluated by comparing principal component analysis (PCA) and uniform manifold approximation and projection (UMAP) distributions before and after integration.

### 2.2. Immune infiltration analysis

In the bulk RNA-seq data, the relative abundances of 22 immune cell subsets were estimated using the Cell type Identification By Estimating Relative Subsets Of RNA Transcripts (CIBERSORT) algorithm, and single-sample gene set enrichment analysis was employed to calculate enrichment scores for immune-related gene sets. Differences between groups were assessed by Wilcoxon rank-sum test, and global infiltration patterns were visualized via hierarchical clustering (Euclidean distance, Ward’s method).

### 2.3. Single-cell clustering and annotation

Highly variable genes from single-cell data were subjected to PCA, and the top 20 principal components were used to construct a k-nearest neighbor graph. Clusters were identified with the Louvain algorithm and visualized with UMAP. Clusters were annotated into 6 major cell types – endothelial cells, oligodendrocytes, oligodendrocyte precursor cells, astrocytes, microglia, and neurons, based on canonical marker genes (e.g., CLDN5, PLP1, PDGFRA, GFAP, C1QA, SNAP25).

### 2.4. Phenotype-associated subset identification

Scissor integrates bulk phenotypic labels with single-cell transcriptomic profiles through sparse regression modeling to identify phenotype-associated cells without direct expression matrix merging.

### 2.5. Co-expression module detection and candidate gene selection

Expression data for oligodendrocytes and astrocytes were used to construct weighted gene co-expression networks with hdWGCNA. Modules were identified and module eigengenes calculated. Modules most associated with VaD phenotype were intersected with bulk differentially expressed genes to nominate candidate regulatory genes.

### 2.6. Pseudotime trajectory analysis

Monocle3 was used to reconstruct developmental trajectories of oligodendrocytes and astrocytes based on differentially expressed genes. Early and late developmental states were defined, and expression dynamics of candidate genes were profiled across pseudotime to elucidate their roles in disease progression.

### 2.7. Functional enrichment analysis

Differentially expressed genes and co-expression module gene sets were analyzed with the clusterProfiler package for Gene Ontology (GO) and Kyoto Encyclopedia of Genes and Genomes (KEGG) enrichment. The enrichGO and enrichKEGG functions performed hypergeometric tests on biological processes, molecular functions, cellular components, and pathways, using adjusted *P* < .05 as the significance threshold. Results were visualized as bubble plots (bubble size = gene count; color intensity = significance). Additionally, gene set enrichment analysis was conducted on ranked gene lists using the MSigDB Hallmark gene sets; enrichment scores and significance (adjusted *P* < .05) were determined, and results displayed with ridge plots for comparative pathway activation.

### 2.8. Molecular docking validation

To evaluate binding affinities and interaction modes between candidate small molecules and the ELF2 protein, we employed AutoDock Vina v1.2.2 – an open-source protein–ligand docking tool (http://autodock.scripps.edu/). Small-molecule structures were retrieved from the PubChem Compound database (https://pubchem.ncbi.nlm.nih.gov/) and converted to PDBQT format using AutoDockTools. The ELF2 protein coordinates were obtained from the Protein Data Bank (https://www.rcsb.org/) and prepared similarly. Molecular docking was conducted as an exploratory analysis intended for hypothesis generation rather than validation of pharmacological efficacy.

All protein and ligand files were processed in AutoDockTools: water molecules were removed, polar hydrogens were added, and Gasteiger charges were assigned. A cubic docking grid box (e.g., 30 Å × 30 Å × 30 Å with 0.05 nm spacing) was centered on the predicted active site to encompass the binding pocket. For each ligand–protein pair, multiple docking runs were conducted, and the conformation with the lowest predicted binding energy was selected for further analysis and visualization.

### 2.9. Computational environment

All analyses were conducted in R (version 4.2.1) on a Linux-based workstation. Key R packages and versions used include: Seurat v4.1.2, CIBERSORT R script v1.03, GSVA v1.46.0, scissor v0.2.0, hdWGCNA v1.1.1, Monocle3 v1.2.9, and clusterProfiler v4.6.1. AutoDock Vina v1.2.2 was employed for molecular docking, installed via the standard binary distribution.

## 3. Results

### 3.1. Distinct immune heterogeneity in VaD

**Figure 1.**
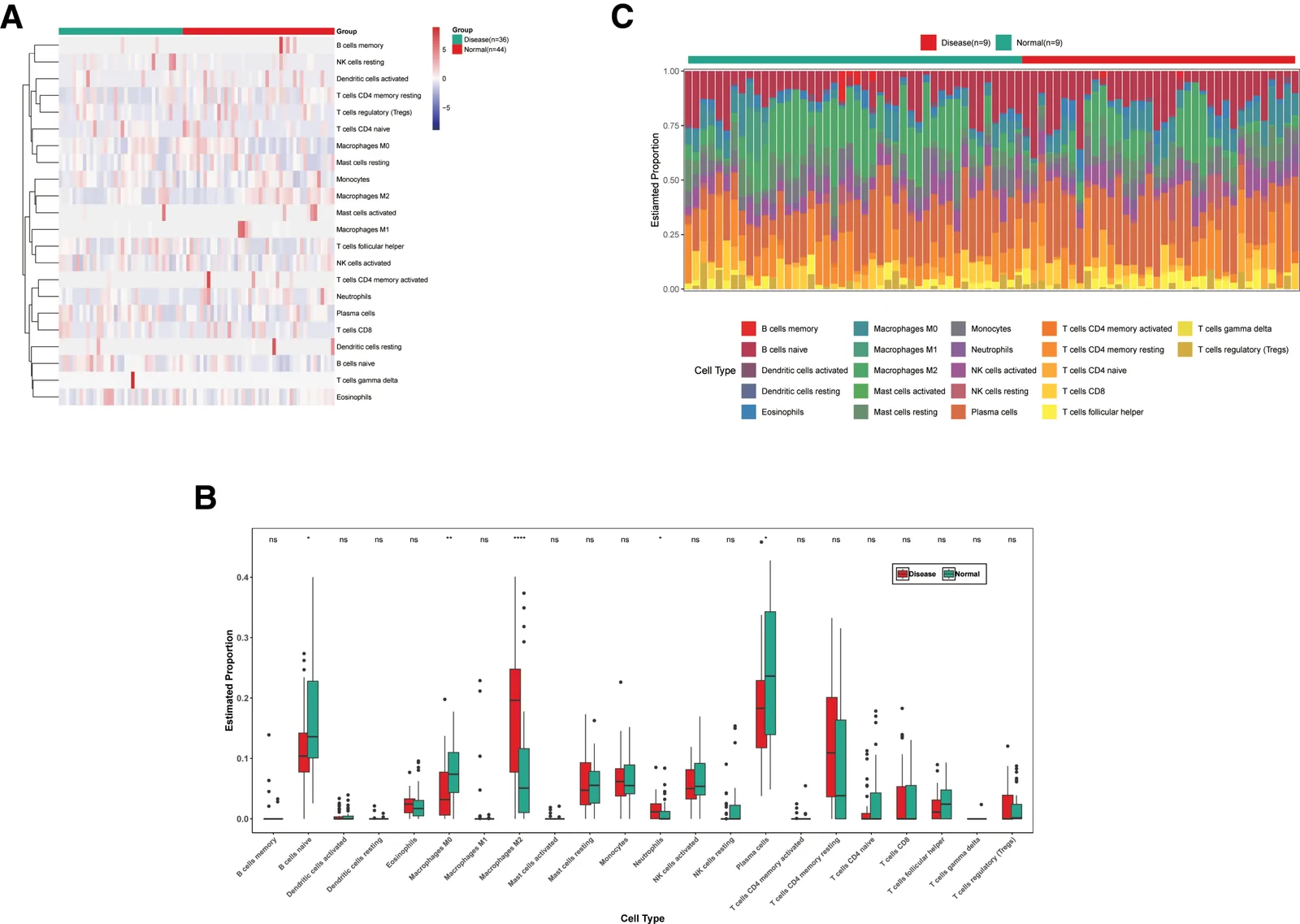
Immune cell Infiltration profiles in VaD and control brain tissues. (A) Heatmap of the relative abundances of 22 immune cell subsets in 18 samples from GSE122063 (green bar: control; red bar: VaD), with hierarchical clustering (Euclidean distance, Ward’s method). Darker colors indicate higher cell proportions. (B) Boxplots comparing the abundance distributions of each immune cell subset between control and VaD groups. The horizontal line denotes the median; the box spans the interquartile range (IQR); whiskers extend to observations within 1.5 × IQR; dots represent outliers. Asterisks indicate significance by Wilcoxon rank-sum test (*P < .05; ****P < .01; ***P < .001). (C) Stacked bar chart showing the relative proportions of immune cell populations in each sample. VaD samples exhibit a general increase in pro-inflammatory cells (e.g., M1 macrophages, neutrophils, activated dendritic cells) and a decrease in anti-inflammatory/regulatory cells (e.g., M2 macrophages, resting CD4+ memory T cells, regulatory T cells). IQR = interquartile range, VaD = vascular dementia.

By mining sequencing data of VaD brain tissue from the GEO database (sample details in Table 1), we performed immune cell deconvolution on GSE122063 bulk RNA-seq data. CIBERSORT analysis of 22 immune subpopulations, followed by hierarchical clustering (Euclidean distance, Ward’s method), revealed that VaD samples exhibit a pro-inflammatory bias – with increased M1 macrophages, neutrophils, and activated dendritic cells – and a concomitant reduction in anti-inflammatory/regulatory populations, including M2 macrophages, resting CD4+ memory T cells, and regulatory T cells (Fig. 1A). Wilcoxon rank-sum tests confirmed significant up-regulation of neutrophils (*P* < .001), M1 macrophages (*P* = .002), monocytes (*P* = .03), and activated dendritic cells (*P* = .02), alongside down-regulation of M2 macrophages (*P* = .01), resting CD4+ memory T cells (*P* = .015), memory B cells (*P* = .04), and regulatory T cells (*P* = .02) in VaD versus control (Fig. 1B). Stacked bar plotting of single-sample gene set enrichment analysis scores further illustrated a disrupted pro- versus anti-inflammatory balance in VaD (Fig. 1C). These findings indicate that localized hyperinflammation, driven by enhanced infiltration of pro-inflammatory cells and loss of immunoregulatory populations, may play a pivotal role in VaD pathogenesis.

**Table 1:** Dataset details.

ID	Data type	Information	Numbers
GSE122063	Microarray	Brain tissue from VaD and non-VaD individuals	N = 80 (Normal = 44; Disease = 36)
GSE282111	10 × Genomics single-cell RNA-seq	Brain tissue from VaD and non-VaD individuals	N = 8 (Normal = 4; Disease = 4)

RNA-seq = RNA sequencing, VaD = vascular dementia.

### 3.2. Neuroglial–vascular cell imbalance

**Figure 2.**
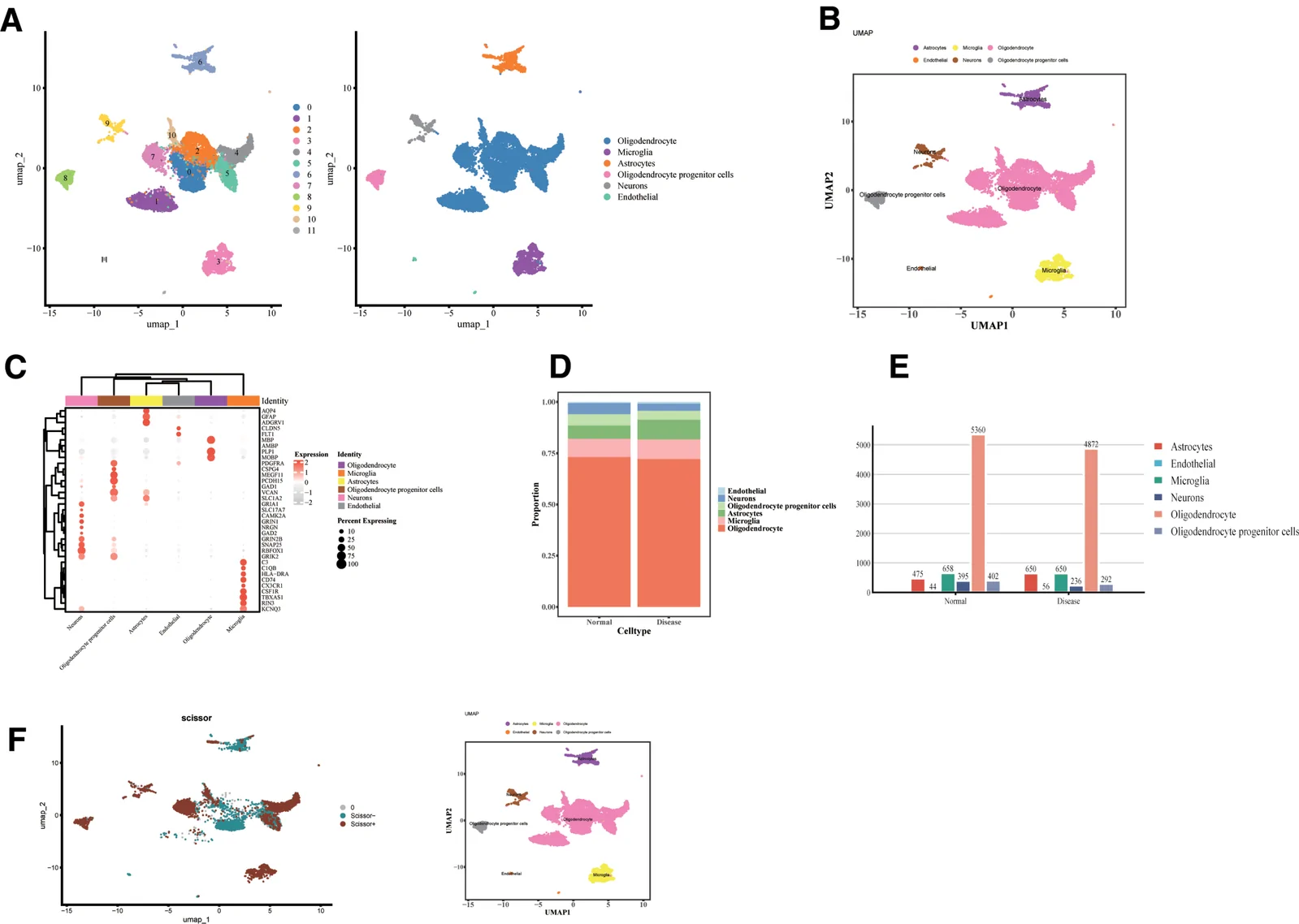
Single-cell RNA-seq clustering and cell type annotation. (A) Louvain clustering based on PCA and UMAP dimensionality reduction consolidated the initial 12 clusters into 6 major cell populations. Left: cells colored by cluster ID. Right: cells colored by annotated cell type (endothelial cells, oligodendrocytes, oligodendrocyte precursor cells, astrocytes, microglia, neurons). (B) UMAP feature plots showing the specific expression of canonical marker genes across cell populations: CLDN5 (endothelial cells), PLP1 (oligodendrocytes), PDGFRA (oligodendrocyte precursor cells), GFAP (astrocytes), C1QA (microglia), and SNAP25 (neurons). (C) Dot plot alongside a hierarchical clustering heatmap: dot size represents the fraction of cells in each population expressing a given marker, and color intensity reflects the average expression level, jointly illustrating the transcriptional signatures of the 6 cell types. (D) Stacked bar chart of the relative abundances of the 6 annotated cell types in control (Normal) versus VaD groups. (E) Comparison of absolute cell counts for each cell type between control and VaD groups, with group-wise differences assessed by Wilcoxon rank-sum tests (*P < .05; ****P < .01; ***P < .001). (F) Identification of cell subsets associated with the VaD phenotype using the Scissor algorithm. Left: UMAP distribution of Scissor^+^ cells (positively correlated with VaD, green) and Scissor^−^ cells (negatively correlated with VaD, brown). Right: integration of the 6 cell type annotations with Scissor classification, highlighting the enrichment of disease-associated cells within each cell type. PCA = principal component analysis, RNA-seq = RNA sequencing, UMAP = uniform manifold approximation and projection, VaD = vascular dementia.

Extending our analysis to single-cell resolution (GSE282111), we employed Seurat to perform quality control (200–5 000 genes per cell; <10% mitochondrial content), LogNormalize scaling, and identification of highly variable genes. Dimensionality reduction (PCA, UMAP) and Louvain clustering (resolution = 0.5) yielded 12 preliminary clusters, which were consolidated into 6 major cell types based on canonical markers: endothelial cells (CLDN5), oligodendrocytes (PLP1), oligodendrocyte precursor cells (PDGFRA), astrocytes (GFAP), microglia (C1QA), and neurons (SNAP25; Fig. 2A–C). Proportion analysis revealed oligodendrocytes as the dominant population (~70%), followed by OPCs, astrocytes, microglia, endothelial cells, and neurons (Fig. 2D). Compared to controls, VaD samples showed significant reductions in oligodendrocytes (*P* = .008), astrocytes (*P* = .020), and endothelial cells (*P* = .030), whereas microglia and OPCs displayed nonsignificant upward trends (Fig. 2E). These results uncover a marked neuroglial–vascular cell imbalance in VaD, particularly oligodendrocyte loss and astrocyte/endothelial dysfunction that likely contributes to disease mechanisms.

### 3.3. Phenotype-associated cell subsets by Scissor

We integrated bulk phenotype labels (GSE122063) with single-cell expression profiles (GSE282111) using the Scissor algorithm (logistic regression with batch adjustment, false discovery rate [FDR] < 0.05). This approach automatically classifies cells into positively (Scissor^+^) or negatively (Scissor^−^) disease-associated subsets. In UMAP space (Fig. 2F), Scissor^+^ cells (brown) clustered primarily within oligodendrocyte, astrocyte, and endothelial compartments, indicating a strong positive association with the VaD phenotype, whereas Scissor^−^ cells (cyan) localized predominately to microglia and OPC clusters, marking a negative association; unannotated cells (gray) were scattered, reflecting minimal correlation. This distribution underscores the critical involvement of neuroglial and vascular cells in VaD pathogenesis.

### 3.4. Molecular diagnostic model construction

**Figure 3.**
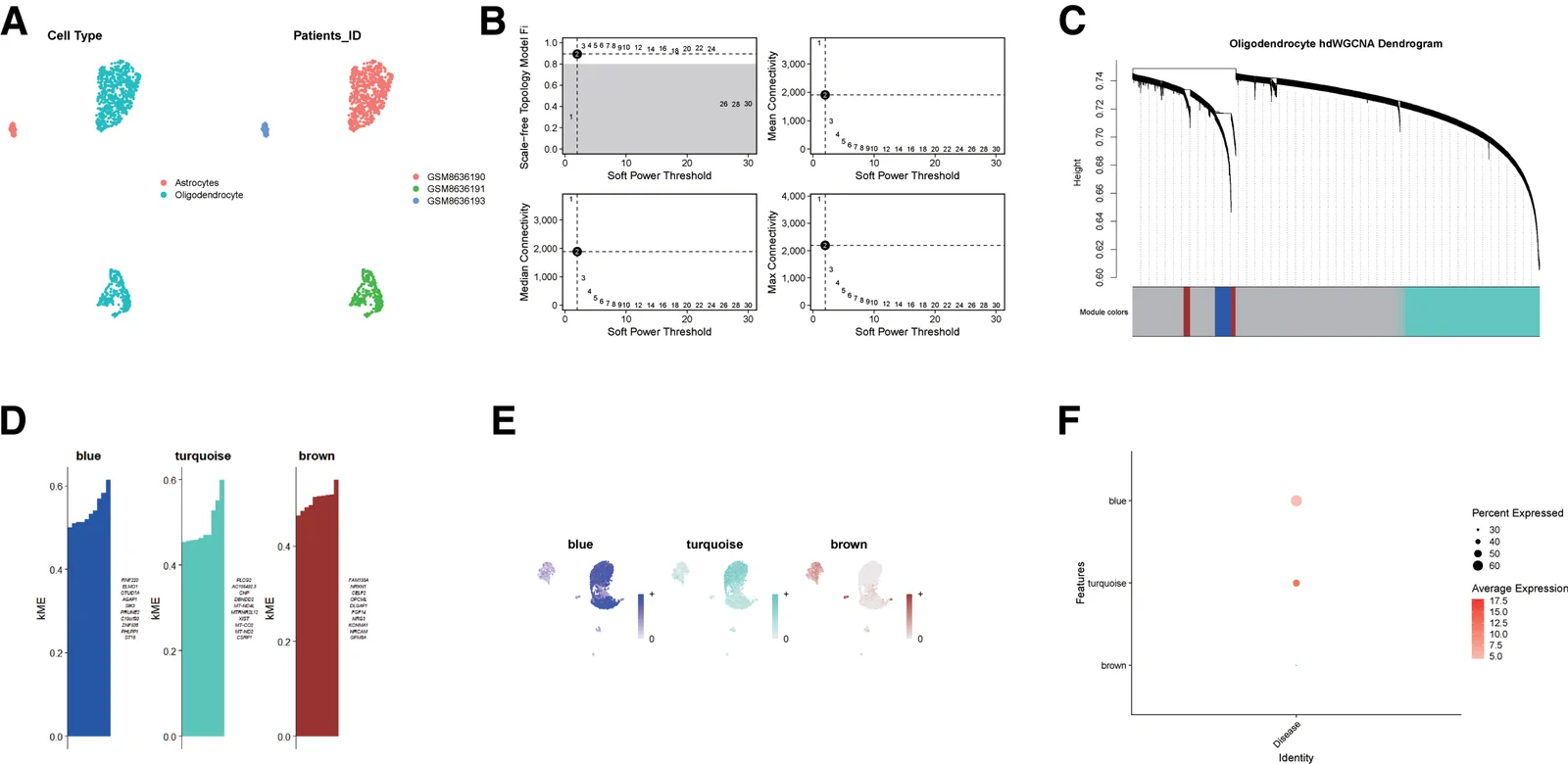
hdWGCNA analysis and gene module identification. (A) UMAP projection highlighting oligodendrocytes (blue) and astrocytes (green). Left: cells colored by annotated cell type. Right: cells colored by patient of origin. (B) Soft-threshold power selection. Left: scale-free topology fit index as a function of soft-power; the dashed line indicates the 0.8 threshold. Right: corresponding mean connectivity for each soft-power. (C) Gene dendrogram and module assignment. Colored bars beneath the tree represent the distinct co-expression modules identified. (D) Module–phenotype correlation barplot showing Pearson correlation coefficients between module eigengenes (blue, turquoise, brown modules) and the VaD phenotype, with significance assessed at FDR < 0.05. The blue and turquoise modules exhibit the strongest correlations. (E) Expression of the blue module eigengene across the UMAP embedding. Regions of higher eigengene expression are indicated by deeper color intensity. (F) Dot plots of hub gene expression within oligodendrocytes and astrocytes. Dot size reflects the proportion of expressing cells, and color intensity represents the average expression level. FDR = false discovery rate, hdWGCNA = high-dimensional weighted gene co-expression network analysis, UMAP = uniform manifold approximation and projection, VaD = vascular dementia.

**Figure 4.**
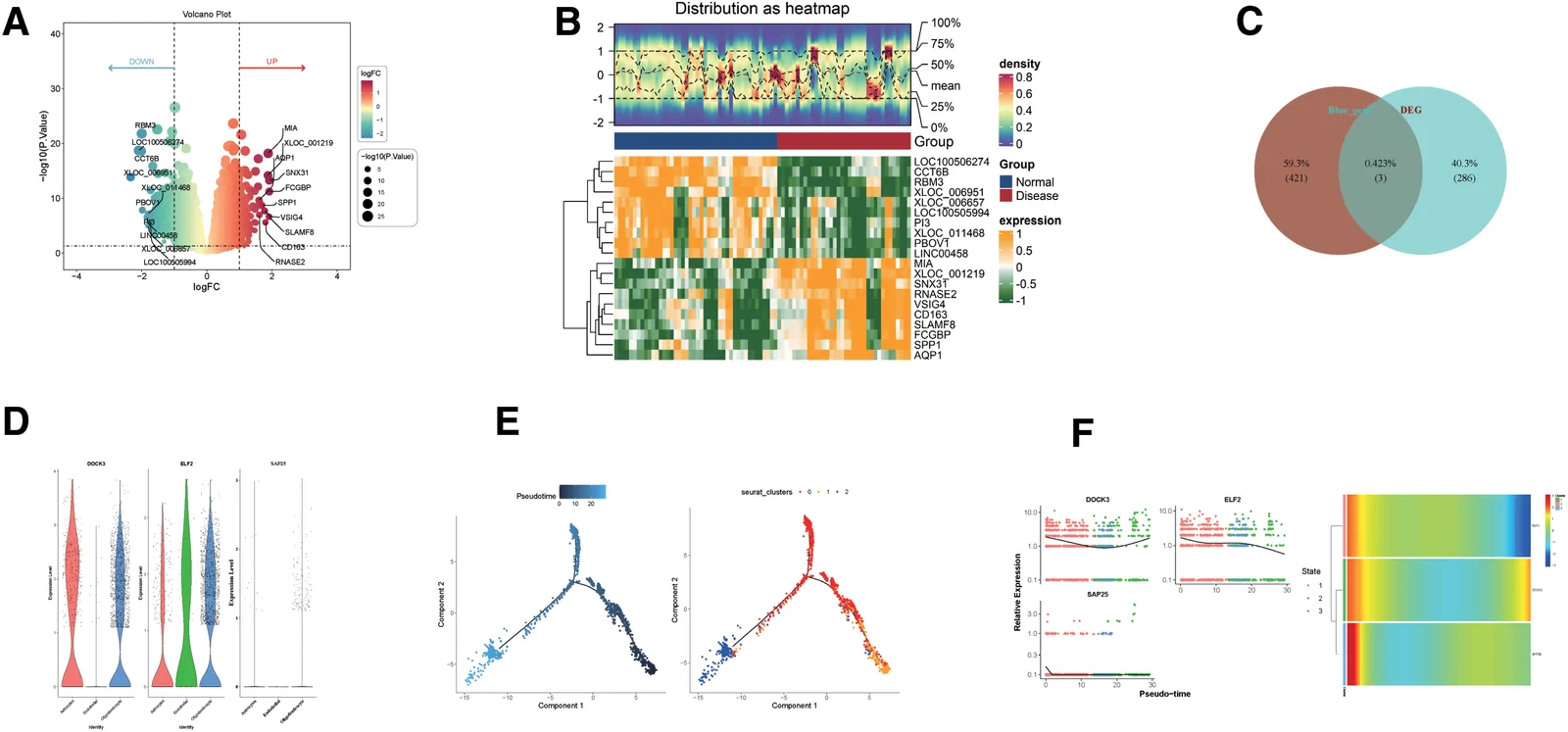
Differential gene identification and single-cell expression of ELF2. (A) Volcano plot of differentially expressed genes (DEGs) between VaD and control samples in GSE122063 (|log_2_ FC| > 1, FDR < 0.05). Red dots indicate upregulated genes; green dots indicate downregulated genes. (B) Heatmap of DEGs across all samples, with hierarchical clustering of samples based on expression similarity, illustrating group-specific gene expression patterns. (C) Venn diagram showing the overlap between bulk DEGs and genes in the hdWGCNA blue module, identifying DOCK3, ELF2, and SAP25 as common candidates. (D) Violin plots of the 3 intersecting genes in VaD versus control samples. Dotted lines and asterisks denote statistical significance (***P < .001). (E) UMAP feature plots displaying the single-cell expression distributions of DOCK3, ELF2, and SAP25, with color intensity reflecting expression level. (F) Left: pseudotime trajectory plots showing dynamic expression of candidate genes along developmental progression; right: corresponding expression heatmap across pseudotime. Only ELF2 exhibits a marked decline over pseudotime, suggesting its potential functional role in VaD progression. DEG = differentially expressed gene, DOCK3 = dedicator of cytokinesis 3, ELF2 = E74 like ETS transcription factor 2, hdWGCNA = high-dimensional weighted gene co-expression network analysis, SAP25 = Sin3A associated protein 25, UMAP = uniform manifold approximation and projection, VaD = vascular dementia.

Focusing on oligodendrocytes and astrocytes, cell types highly abundant in VaD tissues and exhibiting strong regulatory potential, we applied hdWGCNA to their feature genes, identifying 3 co-expression modules (Fig. 3A–D). Correlation analysis highlighted the blue and turquoise modules as most tightly linked to the VaD phenotype; given its higher gene expression, the blue module was chosen as the differential gene set (Fig. 3E, F). Subsequent analysis of VaD bulk RNA-seq data yielded differentially expressed genes (DEGs) visualized by volcano and heat maps (Fig. 4A, B). Intersection of these DEGs with the blue-module genes isolated dedicator of cytokinesis 3 (DOCK3), ELF2, and SAP25 as candidate regulators of immune heterogeneity in VaD (Fig. 4C). Notably, only ELF2 was co-expressed in both oligodendrocytes and astrocytes (Fig. 4D), nominating it as a key mediator of immunological dysregulation.

### 3.5. Pseudotime analysis of developmental dynamics

**Figure 5.**
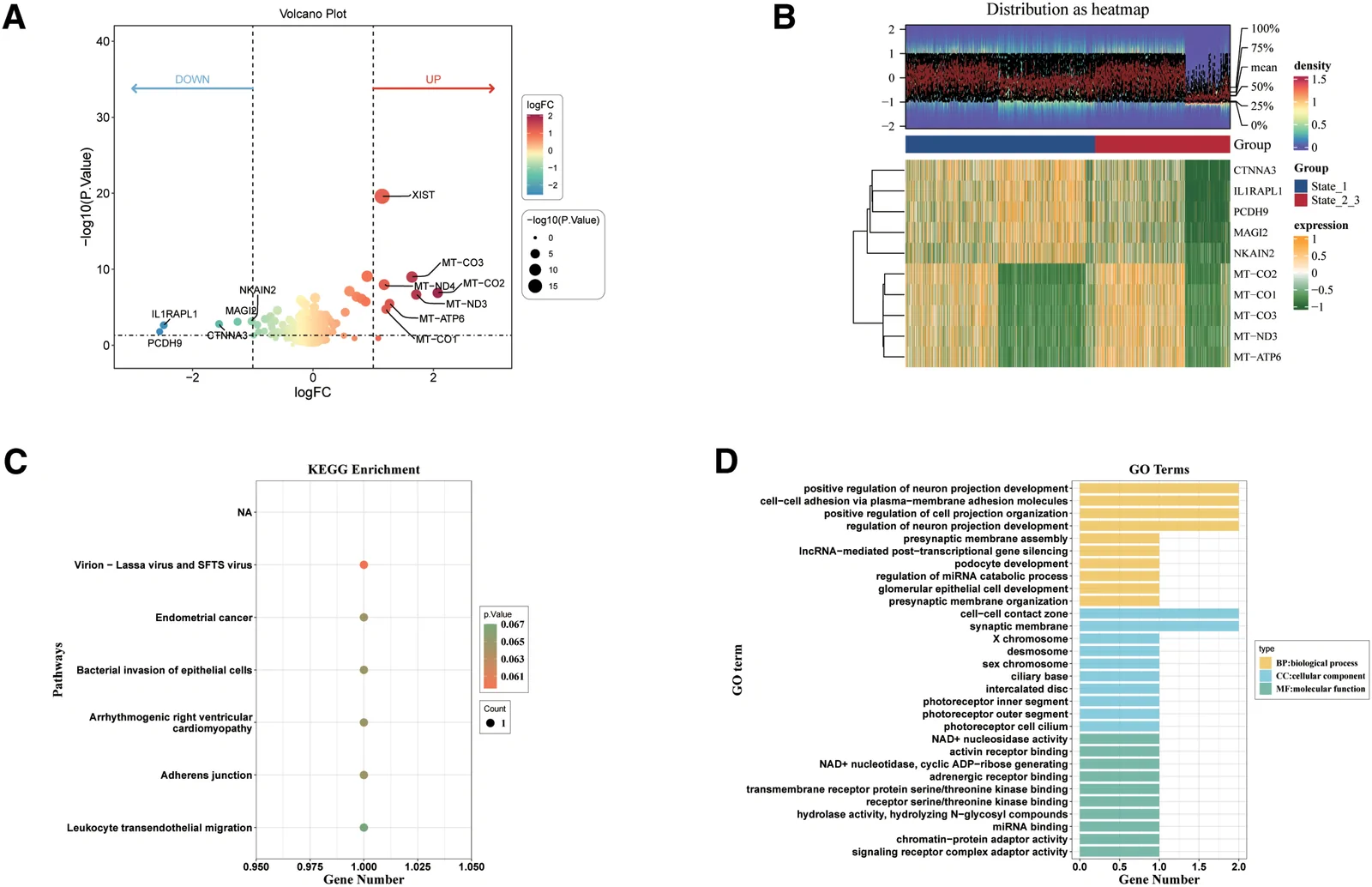
Differential gene identification across developmental states and functional enrichment analysis. (A) Volcano plot of differentially expressed genes between state 1 and state 2/3 cell populations (|log_2_ FC| > 1, FDR < 0.05). Downregulated genes are on the left, and upregulated genes are on the right. (B) Heatmap of these DEGs across all single-cell samples, with both genes and cells hierarchically clustered by expression profile similarity. (C) KEGG pathway enrichment bubble plot: bubble size represents the number of enriched genes, and color intensity (−log_10_P) indicates significance. (D) GO biological process enrichment bar plot showing key processes significantly associated with the DEGs, including neuronal projection development, extracellular matrix remodeling, and apoptosis. DEG = differentially expressed gene, FDR = false discovery rate, GO = Gene Ontology, KEGG = Kyoto Encyclopedia of Genes and Genomes.

We performed pseudotime trajectory analysis on oligodendrocytes and astrocytes, identifying 3 cellular states: state 0 (early) and states 1 to 2 (late differentiation; Fig. 4E). Expression profiling of DOCK3, ELF2, and Sin3A associated protein 25 across these states revealed that only ELF2 exhibited a significant downward trend during VaD-related differentiation (Fig. 4F), suggesting that ELF2 downregulation is associated with disease-related cellular state transitions and immune alterations. Differential expression analysis between state 1 and states 2/3 uncovered widespread gene downregulation in early state and upregulation of factors like XIST in later states (Fig. 5A). Hierarchical clustering heat maps further demonstrated clear grouping patterns, with high intragroup similarity and inter-group divergence (Fig. 5B). KEGG and GO bubble plots revealed enrichment of DEGs in pathways such as viral infection, apoptosis, and pancreatic cancer, as well as biological processes including neuronal projection development and extracellular matrix remodeling (Fig. 5C, D). These data provide mechanistic insight into ELF2’s role in cell fate decisions and signaling networks.

### 3.6. Therapeutic targeting of ELF2

**Figure 6.**
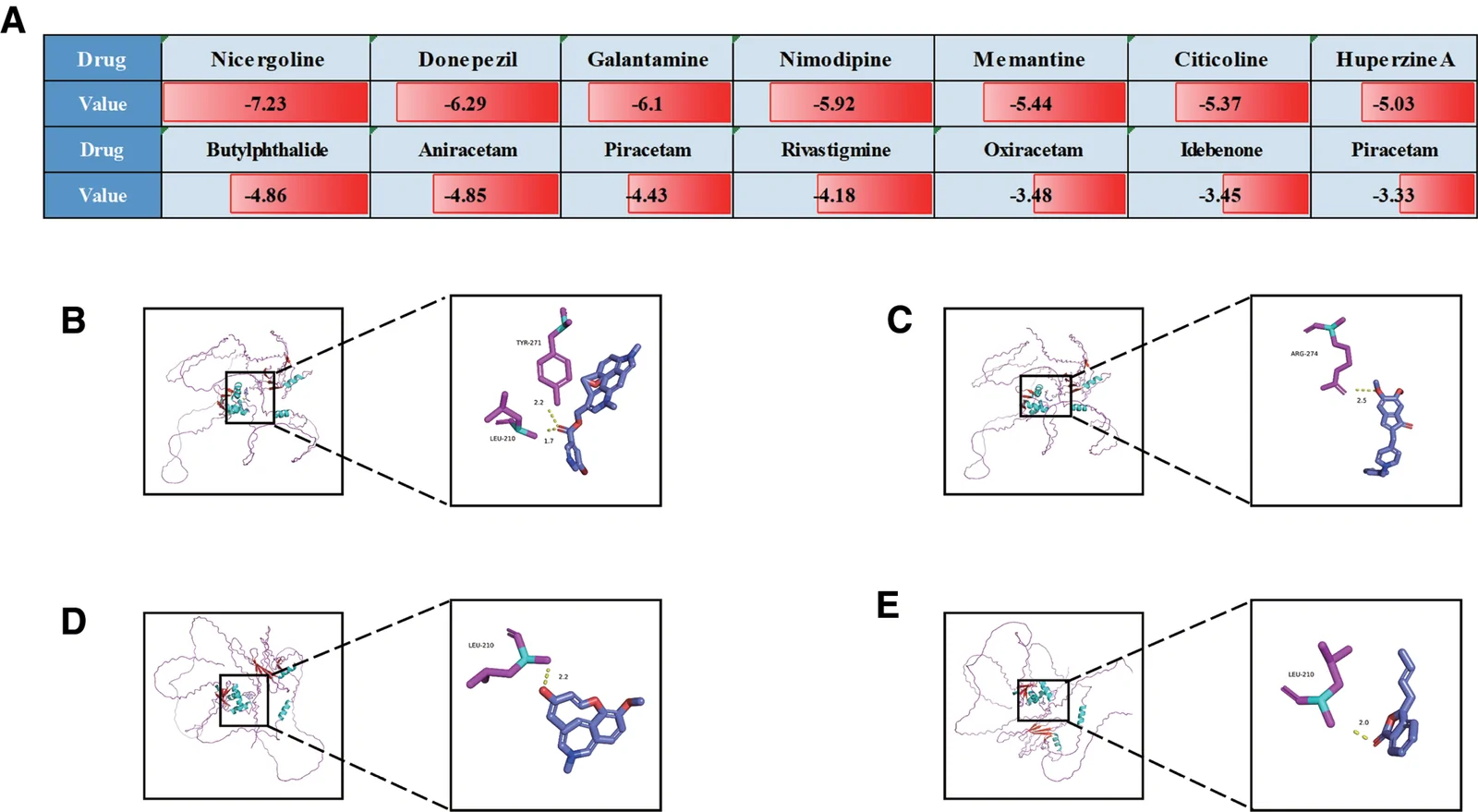
Molecular docking of ELF2 with common single-agent therapies for vascular dementia. (A) Heatmap of predicted binding affinities (kcal/mol) between ELF2 and fifteen single-agent vascular dementia therapies. Lower values indicate more stable binding. The top 3 compounds are nicergoline (−7.23), nimodipine (−6.92), and donepezil (−6.29). Butylphthalide (−4.86) is included as a reference drug noted in the literature for its efficacy and low adverse effect profile. (B) Nicergoline forms multiple hydrogen bonds with His and Glu residues and π–π stacking with Phe, achieving high binding stability. (C) Nimodipine establishes hydrogen bonds with Ser and Asp residues, supplemented by hydrophobic interactions. (D) Donepezil engages Arg and Tyr residues via hydrogen bonding and hydrophobic contacts. (E) Butylphthalide exhibits markedly fewer hydrogen bonds and hydrophobic interactions with ELF2, resulting in weaker affinity, suggesting limited potential for direct ELF2 targeting. ELF2 = E74 like ETS transcription factor 2.

Finally, to explore the translational potential of ELF2, we collected 15 single-agent VaD therapeutics from the literature and performed molecular docking to assess their binding to ELF2 (Fig. 6A). Docking results showed that nicergoline, nimodipine, and donepezil exhibited the lowest binding energies (−7.23,–6.92, and–6.29 kcal/mol, respectively). Nicergoline formed multiple hydrogen bonds with His and Glu residues and π–π stacking with Phe; nimodipine established hydrogen bonds with Ser and Asp alongside hydrophobic interactions; donepezil engaged Arg and Tyr via hydrogen bonds and hydrophobic contacts. In contrast, butylphthalide demonstrated a weaker binding energy (−4.86 kcal/mol) with fewer interactions. These findings identify compounds with predicted binding affinity toward ELF2 and generate hypotheses for future experimental testing, providing a rationale for ELF2-based therapeutic strategies in VaD (Fig. 6B–E).

## 4. Discussion

This study employed an integrative approach, combining bulk RNA sequencing and single-cell RNA-seq data, to dissect the molecular neuropathological features of VaD brain tissue, focusing on immune-mediated inflammation and alterations in glial and vascular cellular components. Our results revealed pronounced immune dysregulation in VaD brains, characterized by an accumulation of pro-inflammatory cells and a relative paucity of anti-inflammatory cells. This finding corroborates prior insights into the role of neuroinflammation in VaD, where chronic hypoperfusion sustains microglia in an M1 pro-inflammatory state, persistently releasing mediators such as IL-1β and TNF-α. This process inflicts neuronal damage and exacerbates cognitive decline. Concurrently, anti-inflammatory M2 microglia and regulatory lymphocytes exhibit impaired functionality, likely due to the ischemic environment and deficient signaling, impeding inflammation resolution. Quantitative analysis of immune cell subtype proportions via single-cell data confirmed an elevated presence of pro-inflammatory macrophage/microglial subsets in VaD tissue, alongside a reduction in immunosuppressive populations. This immune imbalance intensifies damage to neurons and oligodendrocytes and further disrupts the BBB through the release of matrix metalloproteinases and free radicals. Thus, the chronic inflammatory microenvironment in VaD serves as both a disease outcome and a critical driver of progression. Our findings underscore the importance of restoring pro-inflammatory/anti-inflammatory immune balance to halt VaD neurodegeneration, offering a rationale for anti-inflammatory therapeutic strategies.

Additionally, we identified loss or dysfunction of multiple glial and vascular cell types in VaD brain tissue. Single-cell transcriptomic analysis revealed significant reductions in oligodendrocyte and astrocyte populations in the VaD group, accompanied by downregulated expression of associated genes, indicative of impaired myelination. These changes directly correlate with white matter demyelination and cognitive deficits. Differential gene expression analysis further demonstrated widespread downregulation of oligodendrocyte function-related genes, suggesting deficits in their differentiation and survival. In astrocytes, altered expression of genes linked to metabolic support and neurovascular coupling points to structural and functional disruptions in their endfeet. Changes in brain endothelial cell subsets reflect vascular dysfunction and heightened inflammatory signaling, providing cellular evidence for VaD’s vascular pathology and supporting cerebral microvascular protection as a viable intervention strategy.

Using the Scissor algorithm, we identified cell subsets strongly associated with the VaD phenotype from single-cell data, predominantly within microglial and oligodendrocyte lineages.^[30]^ These subsets exhibited a consistent pro-inflammatory molecular profile, highlighting their central role in VaD pathogenesis and offering precise cellular targets for therapeutic intervention.

At the molecular level, cross-analysis of hdWGCNA and differential expression pinpointed key regulatory factors, including the transcription factor ELF2.^[29]^ ELF2 displayed significant downregulation in oligodendrocytes and astrocytes, with pseudotime trajectory analysis revealing a developmental arrest pattern, suggesting its role in mediating impaired glial cell maturation and dysfunction. The ELF2 co-expression network was enriched in pathways related to myelination, apoptosis regulation, and inflammation, reinforcing its multifaceted involvement in VaD pathogenesis. Functional enrichment and molecular docking analyses indicated potential interactions between ELF2 and small-molecule compounds, preliminarily establishing its promise as a drug target.

Although bootstrap-based internal validation supported the stability of the observed immune signatures and ELF2 prioritization, the lack of independent external cohorts remains an important limitation. Future studies incorporating additional multicenter transcriptomic datasets and prospective validation cohorts are warranted. Experimental validation using qPCR, immunostaining, and functional perturbation assays will be necessary to establish the biological role of ELF2 in VaD. The docking results should be interpreted cautiously because they do not account for pharmacodynamics, BBB penetration, or in vivo biological activity.

Through multi-omics integration, this study systematically delineated the immune-inflammatory responses, glial and vascular cellular changes, and molecular regulatory networks in VaD brain tissue, proposing ELF2 as a promising candidate warranting further experimental investigation. ELF2 is associated with transcriptional alterations observed during VaD progression. However, limitations – including small sample size, restricted single-cell data coverage, and the absence of functional validation – necessitate further confirmation in larger cohorts and experimental models. Future research could incorporate proteomics, epigenomics, and spatial transcriptomics to construct a comprehensive molecular atlas of VaD, advancing the clinical translation of precision, target-based interventions.

## Author contributions

**Conceptualization:** Li Liang, Cuiqin Shen, Shiren Huang, Minglu Hu, Jian Zhu.

**Data curation:** Li Liang, Cuiqin Shen, Shiren Huang, Minglu Hu, Jian Zhu.

**Formal analysis:** Li Liang, Cuiqin Shen, Shiren Huang, Minglu Hu, Jian Zhu.

**Writing – original draft:** Jian Zhu.

**Writing – review & editing:** Jian Zhu.
